# 12-Week Inspiratory Muscle Training Improves Respiratory Muscle Strength in Adult Patients with Stable Asthma: A Randomized Controlled Trial

**DOI:** 10.3390/ijerph18063267

**Published:** 2021-03-22

**Authors:** Yi Chung, Ting-Yu Huang, Yi-Hung Liao, Yu-Chi Kuo

**Affiliations:** 1 College of Human Development and Health, National Taipei University of Nursing and Health Sciences, Taipei 112, Taiwan; m9306009@gmail.com; 2 Department of Exercise and Health Science, National Taipei University of Nursing and Health Sciences, Taipei 112, Taiwan; ting850324@yahoo.com.tw (T.-Y.H.); yihung@ntunhs.edu.tw (Y.-H.L.)

**Keywords:** inspiratory muscle training, breathing exercise, asthma, forced vital capacity (FVC), maximal inspiratory pressure (PImax)

## Abstract

This study aims to investigate and compare the effects of conventional breathing exercises and an inspiratory muscle training intervention on clinical symptoms in asthma patients. Sixty asthma patients (40–65 years old) were randomly assigned to either the conventional breathing exercises (BTE) or inspiratory muscle training (IMT) group for a 12-week intervention period. Outcome measurements were performed before and after the intervention, including the spirometry data, maximal inspiratory and expiratory pressures (PImax and PEmax), asthma control test, asthma control questionnaire, six-minute walk test, and three-day physical activity log, were recorded. PImax expressed as % of predicted value controlled for age and gender in healthy subjects (% predicted) increased by 16.92% (82.45% to 99.38%, *p* < 0.05) in the BTE group and by 29.84% (71.19% to 101.03%, *p* < 0.05) in the IMT group. Except for forced vital capacity, which was reduced in the BTE group, all other measured variables improved in both groups, and no statistically significant between-group differences were found. IMT appears to be more effective than breathing exercise intervention in promoting improvements in respiratory muscle strength. IMT may act as an alternative to conventional breathing exercises for middle-aged and elderly asthma patients.

## 1. Introduction

Asthma is one of the most common chronic respiratory diseases, and it affects more than 300 million people worldwide [1]. In Taiwan, the prevalence of asthma is 5.1% in the general population [2]. This disease not only affects the individual and the whole family but also imposes a substantial burden on the healthcare system and the government [3]. Due to the high prevalence of asthma and associated healthcare costs, it is important to identify low-cost alternatives to traditional pharmacotherapy as well as adjunct therapies to supplement traditional treatment strategies for managing asthma and modifying its severity.

Breathing exercises (BTE) and inspiratory muscle training (IMT) are nonpharmacological interventions that improve asthma control. Both exercises have been widely used and are low cost, easy to apply, safe, and considered important adjuncts for asthma treatment [4]. BTE have been routinely used by professionals to control the hyperventilation symptoms of asthma and often appear in educational programs as self-monitoring techniques, which aim to adapt asthma patients to an appropriate breathing pattern with a longer expiration and a lower respiratory rate and thereby reduce hyperventilation and hyperinflation [5]. BTE positively affect pulmonary function, respiratory muscle strength, levels of functional capacity, physical activity, and health-related quality of life (HRQoL) [6], as well as decrease the use of medications, inflammatory and emotional disorders [7].

IMT, a quantitative technique, is a form of resistance exercise designed to strengthen inspiratory muscles by means of providing a resistance during inspiration. The effect of IMT is generated through the inspiratory muscles adapting to overcome the ‘resistance’. The expected role of IMT and its potential role in patients with asthma include an increase in diaphragm thickness and strength, a decrease in exertional dyspnea, and a decrease in the oxygen cost of breathing [8]. There is evidence that IMT also provides benefits, such as pulmonary function, respiratory muscle strength and endurance, levels of functional capacity, physical activity, and HRQoL, as well as reduced emotional disorders, and the use of medical services [4,9]. The addition of IMT, even when applied for a short period, had better rehabilitative effects than respiratory exercise alone [10].

Advantages of non-pharmacological asthma management, such as BTE and IMT, are low-cost alternatives to conventional drug therapy. Although asthma symptoms can be controlled by pharmacotherapy, asthma patients are not found to improve the functional capacity and physical activity adequately [11]. In addition, serious adverse effects of medication could limit the long-term use. The biomechanical alterations induced by asthma may justify the use of BTE and IMT as complements to medications to reduce respiratory difficulties and maximize the outcomes, which might minimize the need for medication and thereby reduce their side effects [12].

However, there has been no consensus regarding the effectiveness of BTE and IMT in asthmatics [3,9] because scientific evidence for the efficacy has been lacking until recently, and there are limited high-quality randomized controlled trials (RCT). In addition, the diagnosis of asthma in older adults has frequently been overlooked because it is frequently masked by other health problems, and the literature regarding exercise in asthma for middle-age and elderly individuals is sparse [13]. In the present study, we aimed to investigate and compare the effects of a 12-week intervention using either BTE or IMT on pulmonary function, respiratory muscle strength, and asthma control (primary outcomes), as well as functional capacity and physical activity (secondary outcomes), in middle-aged and elderly patients with asthma.

## 2. Materials and Methods

### 2.1. Participants

Sixty thoracic outpatients, who were attending a follow-up clinic at a regional medical center in northern Taiwan, were referred by medical professionals and volunteered to participate in this study. They were aged between 40 and 65 years and had been diagnosed with asthma for more than six months by a specialist doctor. Physicians followed the Global Initiative for Asthma (GINA) guideline [14,15] for diagnosis and classification based on a medical history, physical examination, and comprehensive pulmonary function evaluation. The participants were introduced to an “asthma management action plan”, and were not prescribed routine medications throughout the study. The exclusion criteria were as follows: (1) chronic obstructive pulmonary disease or other lung diseases; (2) lung cancer or other terminal tumors; (3) long-term use of oxygen therapy at home; (4) long-term use of a noninvasive positive pressure breathing apparatus; (5) unable to consent and cooperate; (6) active tuberculosis or other infectious diseases; (7) acute pneumonia infection in the past month; (8) untreated hypertension; (9) stroke; (10) presence of any diseases related to the brain, lungs, or cardiovascular system; and (11) ongoing dialysis treatment. The study was conducted according to the guidelines of the Declaration of Helsinki, and approved by the Institutional Review Board (IRB) of Far Eastern Memorial Hospital in Taiwan (protocol code FEMH-106076-E and date of approval on 21 June 2017). All experimental procedures regarding testing and respiratory training were carefully explained to the participants, and written informed consent from participants who met the inclusion criteria was obtained prior to the beginning of the experiment.

### 2.2. Experimental Design and Procedure

**Figure 1:**
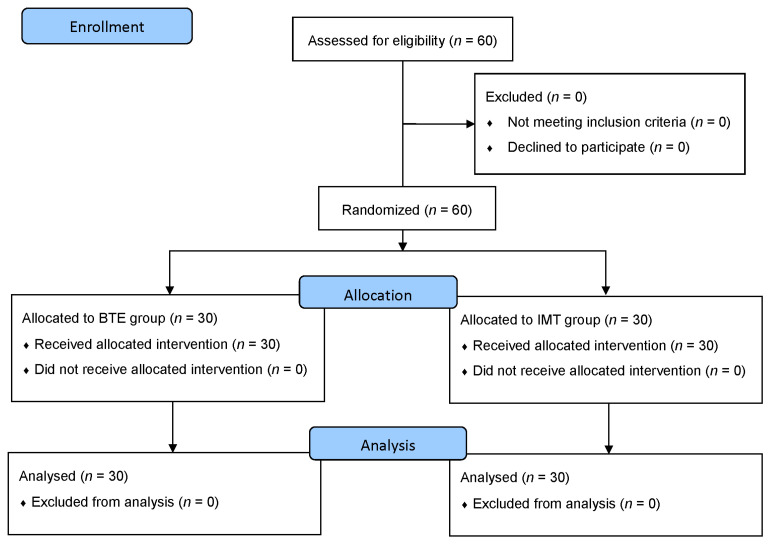
Flowchart of the participants throughout the study (CONsolidated Standards Of Reporting Trials (CONSORT) flow diagram) [20].

The study was a prospective and randomized controlled trial with two arms that compared the non-pharmacological interventions BTE and IMT. A flow diagram of the study is presented in Figure 1. An a priori power analysis with G*Power 3 was performed, using a predefined power of 0.8, an alpha level of 0.05, and an effect size of 0.5 [1]. These parameters lead to a required sample size of at least 38 participants in total (*n* = 19 in each group). We recruited 60 participants, and they were randomly assigned to one of the two intervention groups. After baseline screening and testing, all 60 eligible participants were allocated to the BTE or IMT group by a third person in a blinded manner (i.e., only using the subject number without knowledge of any other identification or baseline test result) using block randomization targeting group sizes of *n* = 30. Participants were concealed from their allocated arm of the study. BTE, IMT, baseline measurement, pre- and post-intervention assessment were all taught and conducted by a single nurse, who is a licensed thoracic nurse practitioner who had special respiratory training, and is familiar with the physiology of BTE and respiratory muscle work.

**Figure 2:**
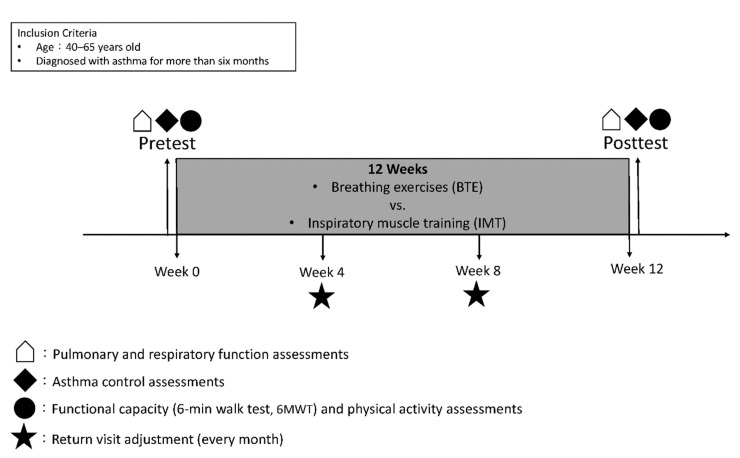
The experimental procedure and timeframe of the study. ⌂: Pulmonary and respiratory function assessments; ♦: Asthma control assessments; ●: Functional capacity and physical activity assessments; ★: Respiratory visit adjustment (in a monthly interval).

During the 12-week program, the BTE group participated in 25-min sessions twice a week (Figure 2). The sessions focused on stimulating nasal and diaphragmatic breathing, increasing the expiratory time, slowing the respiratory flow, and regulating the breathing rhythm. At the beginning of each session, the participants performed exercises to stretch the muscles of the thoracic wall. These exercises were followed by respiratory exercises and exercises to strengthen the abdominal and diaphragmatic muscles. All of the exercises were associated with diaphragmatic breathing and pursed-lip breathing. In pursed-lip breathing, the air is inspired through the nose, causing the abdomen to inflate; then, the air is exhaled through the mouth with semi-open lips. The patients were encouraged to breathe out ‘‘the entire air contents of the lungs’’ [7,16].

Participation in the IMT group required 30 dynamic inspiratory efforts two times per day, five days per week, for 12 weeks. The intensity of each breath was 50–60% of maximal inspiratory pressure (PImax). The participants were instructed to initiate each breath from residual volume and to continue the inspiratory effort until the maximal lung pressure was attained for at least one second. This procedure was repeated three times. A nose clip was worn during all breaths, and the participants were instructed to maintain a low breathing frequency to avoid hyperventilation. The initial training loads were set by the investigators, and all of the inspiratory efforts were trained using a pressure-threshold device (Powerbreathe, HaB International, Southam, UK). The participants were instructed to periodically increase the resistive load such that the completion of 30 breaths approximated the limit of inspiratory muscle tolerance. To ensure that participants adhered to the specific training requirements of the respective training interventions, they reported to the laboratory on a monthly basis, and the respiratory training diaries were completed by them or their family throughout the intervention [17,18,19].

All outcomes were assessed by the same investigator. All the experimental procedures were identical between the pre- and posttest.

### 2.3. Measurements of Pulmonary Function

Forced expiratory volume in one second (FEV1), forced vital capacity (FVC), and inhalation/exhalation flow-rate changes were measured by body plethysmography (MasterScreen Body, Cardinal Health, Höchberg, Germany). The flow accuracy was 0.2–12 L/s ±2%, the box pressure was ±10.2 cmH_2_O (1 kPa) ±2%, and the mouth pressure was ±204.5 cmH_2_O (20 kPa) ±2%. All the tests were repeated three times, from which two sets of data were chosen for this analysis [21,22].

### 2.4. Evaluation of Respiratory Muscle Strength

We used a respiratory muscle pressure meter (MicroRPM, Micro Medical Ltd, Rochester, UK) to determine the PImax and maximal expiratory pressure (PEmax) scores as indicators of respiratory muscle function. The scores for each participant were measured 10 times, in accordance with the American Thoracic Society European Respiratory Society Statement on Respiratory Muscle Testing. Measurements taken during disturbances such as coughing, leakage, and instrument blockage were removed from the analysis. Three measurement values showing less than 5% variance were included for calculation, from which the largest values were recorded as the PImax and PEmax value [23].

### 2.5. Asthma Control Assessments—Asthma Control Test (ACT) and Asthma Control Questionnaire (ACQ)

The ACT is a validated, patient-completed measure of asthma control that includes five questions that assess activity limitations, shortness of breath, nighttime symptoms, use of rescue medication, and patient overall rating of asthma control over the previous four weeks. The questions are scored from 1 (worst) to 5 (best), and the ACT score is the sum of the responses, giving a maximum best score of 25. According to the GINA, an ACT score less than 19 is defined as poorly controlled asthma, and therefore, a score greater than 20 is the optimal cutoff point defining well-controlled asthma [24]. The ACQ has high reliability and is very responsive to changes in asthma control. The scales are scored from 0 to 6 (higher is worse). An ACQ score < 1.5 is considered indicative of controlled asthma, whereas a score > 1.5 is considered indicative of poorly controlled asthma. A clinically effective treatment results in a 0.5-point decrease in the score after an intervention [7].

### 2.6. Six-Minute Walk Test (6MWT), Heart Rate, and Blood Oxygen Saturation

The 6MWT was used to detect participants’ functional capacity by measuring the distance they traveled during the walk test [23]. Considering that the recruited participants did not have regular exercise training experience, we selected this endurance test [25] for our study population because the 6MWT has been used to test functional capacity in the obese population. The 6MWT was performed after the pulmonary function and respiratory muscle strength test in the morning to ensure consistency. For the 6MWT assessment, the participants were encouraged to walk as far as they could during 6 min over a flat 100-feet surface, and for consistency, the researchers used constant verbal cues and positive feedback to encourage the participants to complete the task [26,27]. Meanwhile, heart rate and blood oxygen saturation were measured with a WristOx2^®^ Model 3100 pulse oximeter (Nonin Medical, Inc., Plymouth, MN, USA) during the 6MWT [28]. 6MWT was performed twice to accurately assess exercise performance due to being familiar with 6MWT and the presence of a learning effect, considering the highest value of the six-minute walk distance (6MWD).

### 2.7. Three-Day Physical Activity Log (3-D PAL)

The 3-D PAL was used to estimate energy expenditure by daily physical activity [29]. The participants were divided into retired and employed groups due to different physical activity levels. The employment status was reported by the participants. In the activity log, a day was divided into 96 periods of 15 min each. Each 15-min period over three days, including a weekend day, was quantified in terms of energy costs on a 1 to 9 scale corresponding to a range of 1.0 metabolic equivalent (MET) to 7.8 METs and higher. Then, the researchers calculated the total amount of time spent engaging in moderate physical activity [30].

### 2.8. Statistical Analysis

This study used IBM SPSS Statistics for Windows, Version 20.0 (IBM Corp., Armonk, NY, USA.) to facilitate data processing and analysis. Baseline characteristics of the participants are presented as the mean and standard deviation (mean ± SD) and analyzed by independent t tests and Pearson chi-square tests. The change scores between pre- and post-intervention for each outcome variables in both groups were analyzed using an independent t test. Two-way analysis of variance (two-way ANOVA) was used to evaluate differences between groups. The level of statistical significance was set at *p* < 0.05.

## 3. Results

### 3.1. Baseline Characteristics of the Participants

Both groups had similar demographic and clinical characteristics at baseline (Table 1). Table 2 presents descriptive statistics between the two groups. The results showed that there were more female (*n* = 45, 75%) than male participants (*n* = 15, 25%). The largest proportions of the subjects were accounted for by the groups of 50–59-year-olds (*n* = 25, 41.7%) and groups with BMI between 18.5 and 24 (*n* = 21, 35%), body fat greater than 25%, and moderate asthma (*n* = 56, 93.3%).

### 3.2. Pulmonary Function

**Figure 3:**
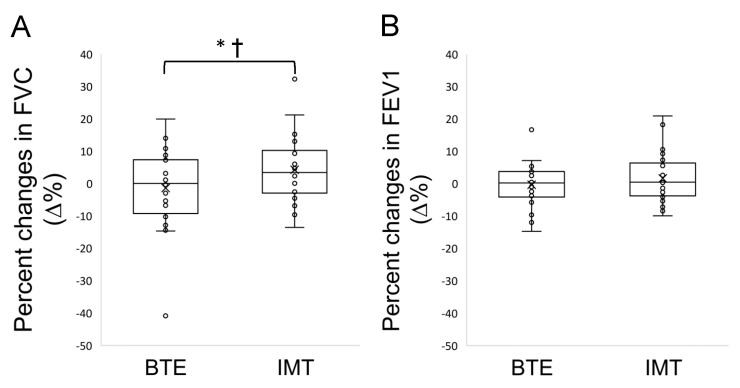
Effects of the breathing exercises (BTE) and inspiratory muscle training (IMT) on the forced vital capacity (FVC) (**A**) and forced expiratory volume in one second, FEV1 (**B**). Values are mean ± SD. * denotes the significant differences between the BTE and IMT groups. † denotes the significant “Treatment (Group) × Time” interaction. (*p* < 0.05).

The FVC results showed a significant interaction (*p* = 0.045). The main effects for “Time” (*p* = 0.312) and “Group” (*p* = 0.147) were not significant (Table 3). For FEV1, there was no effect of “Time” (*p* = 0.498) or “Group” (*p* = 0.505) or “Treatment × Time” interaction (*p* = 0.263). In the IMT group, the FVC (% predicted) increased by 4.25% (from 79.38% ± 13.26 to 83.63% ± 11.54, *p* < 0.05), whereas it did not significantly change in the BTE group (Figure 3).

### 3.3. Respiratory Muscle Strength

**Figure 4:**
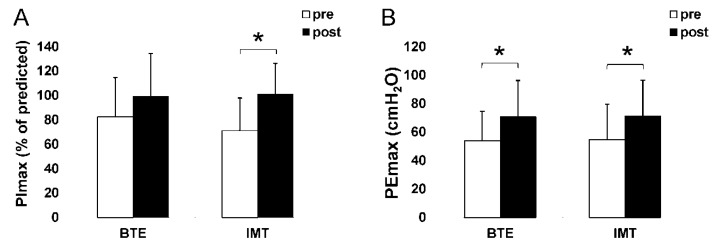
Effects of the breathing exercises (BTE) and inspiratory muscle training (IMT) interventions on the PImax (**A**) and PEmax (**B**). Values are mean ± SD. Pretest is illustrated as a white bar, while posttest is illustrated as a black bar. * denotes the significant differences between pre- and posttest within BTE or IMT group. (*p* < 0.05).

The PImax was expressed as % of the predicted normal value controlled for sex and age [31]. The PImax results showed a significant “Treatment × Time” interaction (*p* < 0.001) and a main effect for “Time” (*p* < 0.001). The main effect for “Group” was not significant (*p* = 0.527). The PImax in the IMT group, % predicted PImax increased by 29.84% (from 71.19 ± 26.66% to 101.03 ± 25.04%, *p* < 0.05) (Figure 4 and Table 3). No statistically significant differences were observed between the two groups.

In the PEmax test, the “Treatment × Time” interaction was not statistically significant (*p* = 0.9), nor was the main effect for “Group” (*p* = 0.918). In contrast, a main effect was observed for “Time” (*p* < 0.001). The PEmax of the BTE group increased from 53.87 ± 20.72 to 70.67 ± 25.5 cmH_2_O and that of the IMT group rose from 54.7 ± 24.83 to 71.07 ± 25.26 cmH_2_O (Figure 4 and Table 3).

### 3.4. Asthma Control

The ACT results showed a main effect for “Time” (*p* = 0.001), and there was no difference between the groups (*p* = 0.927) and no interaction (*p* = 0.437). The ACT scores rose from 21.43 ± 2.06 to 22.06 ± 1.86 in the BTE group and from 21.3 ± 2.95 to 22.3 ± 2.1 in the IMT group.

Regarding the ACQ, the results showed a main effect for “Time” (*p* < 0.001), and there was no difference between the groups (*p* = 0.883) and no interaction (*p* = 0.466). The ACQ scores decreased from 1.15 ± 0.44 to 1.02 ± 0.43 in the BTE group and from 1.2 ± 0.52 to 1 ± 0.41 in the IMT group (Table 3).

### 3.5. 6MWT

The 6MWT was used to assess functional capacity in this study. The “Treatment × Time” interaction was not statistically significant for the 6MWD (*p* = 0.237), and there was no main effect for “Group” (*p* = 0.741). In contrast, a main effect was observed for “Time” (*p* < 0.001). Specifically, the “Treatment × Time” interaction was explored to check for group differences over time and the time differences within groups. No significant differences were observed between the groups after the intervention. The BTE group presented an increase from 520.17 ± 74.8 to 545.1 ± 71.44, and the IMT group also showed an increase from 508.67 ± 71.92 to 545.06 ± 60.3 in the 6MWD. There was no effect for “Time” (*p* = 0.076) or “Group” (*p* = 0.524) or an interaction (*p* = 0.318) on heart rate assessed during the 6MWT. The blood oxygen saturation during the 6MWT showed a main effect for “Time” (*p* = 0.003), and there was no difference between the groups (*p* = 0.440) and no interaction (*p* = 1).

### 3.6. Physical Activity

The 3-D PAL was used as an indicator of physical activity in this study. The participants were divided into a retired group and an employed group for comparison. Among those in the BTE group, 13 participants reported retirement, and 17 did employment while in the IMT group, 12 participants were retired, and 18 were employed. In the retired group, the “Treatment × Time” interaction was not statistically significant (*p* = 0.587), and there was no main effect for “Group” (*p* = 0.765). In contrast, a main effect was observed for “Time” (*p* = 0.001). The 3-D PAL in the retired group showed increases from 38.15 ± 5.67 to 39.51 ± 5.06 in the BTE group and from 37.36 ± 3.95 to 39.15 ± 4.37 in the IMT group. In the employed group, the results showed a main effect for “Time” (*p* = 0.001), there was no difference between the groups (*p* = 0.467), and there was no interaction (*p* = 0.462). The 3-D PAL in the employed group showed increases from 44.01 ± 4.57 to 45.34 ± 8.07 in the BTE group and from 45.3 ± 8.06 to 47.33 ± 8.51 in the IMT group (Table 3).

## 4. Discussion

This study compared the effects of 12 weeks of BTE and IMT interventions on measures of clinical efficacy in middle-aged and elderly individuals with asthma. Our results suggested that IMT improves respiratory muscle strength as reflected by increased PImax. In addition, BTE and IMT resulted in similar positive effects on functional capacity and physical activity. Interestingly, the PImax recorded in middle-aged and elderly patients with stable asthma in this study, was only 72 to 82% predicted. This suggests that there was a need to improve the strength of respiratory muscles in this patient population. 

Although much difference exists in the number of sessions for the two intervention groups, the total intervention duration is similar in both groups. In addition, the IMT protocol we adopted in this study is a representative protocol involving training for 30 breaths twice daily set to 50% PImax [8].

### 4.1. Primary Outcome

Although the FVC statistically increased in the IMT group, it failed to reach the minimal clinically important difference (MCID) [32]. Accordingly, the pulmonary function, FVC and FEV1, did not change in both BTE and IMT groups. Previous studies measuring static and dynamic lung volumes in asthmatics pre- and post-IMT have demonstrated controversial results, as some studies have shown increases in FVC and FEV1 [33,34,35] while others observed no changes in FVC or FEV1 [27,36,37,38,39,40,41,42]. These differences may depend on duration and type of IMT, as well as participant population. Our results in FVC and FEV1 were consistent with the later studies [27,36,37,38,39,40,41,42]. Compared to our study, the former studies used higher training intensity (≤80% PImax vs. our 50–60% PImax) [33], longer training period (30 min vs. our 10 min) [33], and duration (6 months vs. our 3 months) [33], as well as younger participants (40.5 and 21.9 vs. our 55.1 years) [33,34,35]. 

In contrast, the impact of IMT on asthma almost uniformly demonstrated an improvement in inspiratory muscle strength (PImax) and endurance [33,36,37,38,39,40,41,42] that were in line with our findings. These improvements were observed across a wide spectrum of populations with varying degrees of asthma severity [33,34,35,36,37,38,39,40,41,42]. Our findings suggest that increases in inspiratory muscle strength may mediate the improvement of functional capacity and physical activity in asthma patients. Moreover, the interventions themselves were also varied. Therefore, despite the fact that inspiratory muscles could adapt differently depending on the techniques and type of demands placed upon them, the variability in methods may not be important as long as the stress on the respiratory muscle is sufficient. This supports the efficacy of IMT as a management strategy for asthma that could reduce the severity of the disease and be a complementary strategy to traditional treatments.

Our results showed that both BTE and IMT increased respiratory muscle strength, PEmax (PImax only in IMT), which is comparable to the results from previous studies investigating different age populations [8], including BTE programs in elderly asthma patients (>65 years) for 16 weeks [16], asthmatic adults for 12 weeks [43], and moderately asthmatic children (6–17 years) for 6 weeks [44]. Similarly, IMT was reported to cause significant increases in respiratory muscle strength for 6 weeks [36] and 12 weeks [40]. A meta-analysis was carried out in favor of increasing PImax during IMT, but PEmax results were controversial [4]. There are three possible explanations. (1) IMT may elicit subtle improvements in airway diameter and airflow limitation during exercise, thereby reducing dynamic hyperinflation and increasing exercise capacity [45]. The rising mechanical action on the inspiratory muscles including external intercostals, which affirmatively have an accessory participation in expiration, caused greater thoraco-abdominal mobility, as a result of consequent mechanical reorganization of all of the muscles involved in respiration. Moreover, during IMT and BTE, inspiration and expiration are active throughout the respiratory cycle, fostering muscle function optimization, as evidenced by increased muscle strength [42]. (2) The inspiratory pressures imposed on the airways may lead to reduced lung hyperinflation, and thus facilitate the action of the inspiratory muscles. Lung hyperinflation, which causes an increase in final expiratory volume, is a characteristic of asthma. It happens because of the premature closing of small-caliber airways that increases the activity of the respiratory muscles at the end of exhalation [8]. In addition, IMT may offset the functional weakening of the inspiratory muscles that arises due to dynamic hyperinflation. Otherwise, that would lead to the recruitment of high-force-generating highly fatigable accessory muscle fibers, which worsen the development of respiratory muscle fatigue. The reduction of inspiratory muscle fatigue may also have contributed to the improvement in exercise time to the limit of exercise tolerance by delaying the onset of the inspiratory muscle metaboreflex in asthma patients [45]. (3) Another reason may be the loading intensity. IMT involves resistance training on respiratory muscles while BTE is simply to educate patients with appropriate breathing patterns without resistance loads. IMT has been shown to increase diaphragm thickness and increase the proportion of type I fibers and the size of the type II fibers in the accessory inspiratory muscles. Increases in muscle fiber cross-sectional area could reverse or delay the deterioration of inspiratory muscle function and improve inspiratory muscle economy. Consequently, IMT could stimulate hypertrophy of the diaphragm and accessory inspiratory muscles, facilitating more force for a given level of respiratory muscle drive [8]. Therefore, the increase in inspiratory muscle strength in the IMT groups in the present study likely occurred because the respiratory mechanics of the patients was favored and a reduction in respiratory work appeared [46].

BTE is performed using an appropriate breathing pattern to reduce hyperventilation and hyperinflation, thereby normalizing CO_2_ levels, which may reduce bronchospasm and breathlessness [3]. It is related to a positive effect on the effectiveness of air released during expiration. The releasing of air during expiration can be maximized using respiratory muscles correctly [47]. Moreover, these changes in lung volume seem to relax the smooth muscle in the airway [48]. In an asthma animal model, the airway resistance is reduced when the animals are ventilated with a greater tidal volume and lower breathing frequency, compared with those animals ventilated with a smaller tidal volume and higher breathing frequency [49]. However, this hypothesis remains to be confirmed in humans.

The ACT and ACQ were used to investigate asthma control in the subjects. Our results demonstrated that BTE and IMT both had statistically significant effects on asthma control. However, the change in ACT and ACQ score in this study did not reach the respective MCID. The baseline ACT and ACQ scores of participants showed that their asthmatic condition was well controlled prior to the 12-week programs because the ACT scores were all above 19, and ACQ scores were all around 1. The high baseline characteristics in asthma control may indicate a potential ceiling effect, and consequently limit the training effect in this study. In further study, we suggest that recruiting the lower ACT and ACQ participants may be reasonable to expect further improvement. However, our findings showed that both BTE and IMT statistically significantly increase ACT and ACQ scores, which may shed light on the future direction of such studies.

### 4.2. Secondary Outcomes

Functional capacity was similar between the BTE and IMT groups over the course of the study. In the 6MWT, walking distance, blood oxygen saturation, and heart rate can provide indicators of functional capacity, pulmonary gas exchange, and cardiovascular stress, respectively [50]. Our results suggested that both BTE and IMT induced similar increases in the 6MWD, which is consistent with the results of Majewski et al. [26], in which the subjects with bronchial asthma received an 8-week stretching exercise followed by abdominal breathing training. They state that BTE may relieve the uncomfortable symptoms of asthma patients that increased the tolerance for walking during the test [26]. Contrary to our results, Rondinel et al. [27] showed that IMT did not improve functional capacity assessed by the 6MWD, possibly because the subjects had good prior exercise tolerance and no aerobic intervention. In addition, no significant differences in blood oxygen saturation or heart rate were found between the two groups in our study, which is consistent with the results of Pereira et al., who found no significant drop in blood oxygen saturation [50]. Although our data indicated no significant differences in the 6MWD between the two groups, the IMT group increased by 37 m, which tended to surpass the 25-m increase obtained in the BTE group, and also exceed the MCID for 6MWD.

BTE and IMT showed similar results in physical activity. The 3-D PAL was used to determine physical activity, including daily energy expenditure and time spent in moderate- and high-intensity physical activities. Individuals with asthma had lower levels of physical activity than healthy individuals assessed by a physical activity questionnaire (International Physical Activity Questionnaire-Short Form), suggesting that such patients have reduced physical performance stemming from a sedentary lifestyle [51]. 

The retired and employed groups both showed significant differences in 3-D PAL after 12 weeks of BTE and IMT interventions. Specifically, the IMT group tended to show greater increases in their physical activity time than the BTE group did. The adoption of health education programs and professional guidance to improve a patient’s respiratory muscle strength, therefore improve the ventilatory control may be associated with confidence in performing physical activity [48]. Our findings also suggested that the retired group tended to make more progress in physical activity than the employed group. The results are partially consistent with the previous literature [51,52,53]. A possible explanation may be that the amount of physical activity in the retired group did not meet the standard recommended by the Health Promotion Administration, Ministry of Health and Welfare in Taiwan. On the other hand, subjects in the employed group were more likely to have maintained regular physical activity, so they had already reached the standard of physical activity before the intervention, i.e., they had reached a ceiling effect. 

3-D PAL was used to measure the physical activity in older adults previously [54,55], and provide reasonable validity compared with an accelerometry-based monitor [56]. It is also the most used physical activity scale from 1990 to 2009 [57], and there were no clear trends in the degree to which physical activity measured by self-report and direct measures differ [58]. Self-report activity logs require participants to record in real time which provides the most detailed data, and can overcome some limitations of questionnaires, such as less susceptible to recall errors, social desirability bias, and measurement bias [59]. Therefore, we asked the participants to record their physical activity in real time or the next day within the 12-week intervention to eliminate the confounding factor. Moreover, to ensure participants’ adherence, they reported to the laboratory on a monthly basis, and the training diaries were completed by them or their family throughout the intervention. However, we agree that employment might affect the recording of the 3-D PAL in workers. Work-based and leisure time activities mixed may probably dilute the training effect. Nevertheless, the three-day period includes a weekend day and two weekdays, which might balance some occupational activities. 

### 4.3. Limitation

For practical reasons related to the implementation, this study was unable to incorporate a double-blind design of the evaluators and subjects. The participants knew which exercise they performed. Additionally, the non-blind design of the evaluations leads to the possibility of assessment bias. However, the 6MWT was adopted, and walking distance is an objective, observer-independent measure [60]. Moreover, the FVC, FEV1, PImax, and PEmax were measured with quantitative, objective, and monitoring devices. ACT, ACQ, and 3-D PAL are self-reported measures by participants. Furthermore, IMT increased PImax in this study was supported by the related works [8,36]. Confounding factors have been eliminated to the best of our efforts, but there were still some limitations. 

The number of samples in this study was small, so the findings may be difficult to generalize to all asthma patients. Larger samples should be collected in the future. In addition, features of pulmonary function testing can be added into the inclusion criteria to reduce the baseline difference in subject characteristics between groups. 

With a lack of a no-treatment control group, this study may demonstrate the natural course of the condition, but a more active intervention, such as BTE and IMT, may have led to better long-term outcomes [8]. However, we are unaware of any studies that perform a no-treatment control group in an asthma study due to ethics of research involving human subjects: the principles of beneficence.

Last, the long-term effects of the training are still unknown. Whether the training had differential effects based on different training times and training intensities remains to be revealed. In future studies, the effects of the intervention can be followed up for a longer time to be validated as clinical indicators.

## 5. Conclusions

Our findings revealed that representative IMT protocol, involving training for 30 breaths twice daily set to 50% PImax, could increase inspiratory muscle strength, which may mediate the improvement of functional capacity and physical activity in middle-age and elder adults with moderate to severe asthma severity.

Outpatients with moderate-to-severe asthma who participated either in BTE or IMT programs presented similar results regarding pulmonary function, asthma control, functional capacity, and physical activity. However, the participants with IMT appeared to outperform BTE participants in measures of inspiratory muscle strength. These results are relevant in clinical practice to support the benefits of non-pharmacological interventions. The findings suggested significant effects of IMT on respiratory muscle strength in middle-aged and elderly individuals with asthma. IMT provides an alternative treatment for asthma with a more measurable and quantitative approach that may increase the effectiveness of treatments.

## Figures and Tables

**Table 1:** Baseline characteristics of participants in the breathing exercises (BTE) and inspiratory muscle training (IMT) groups.

	BTE	(*n* = 30)	IMT	(*n* = 30)	
	Mean	SD	Mean	SD	*p* Value
Age (year)	55.1	7.72	55.1	6.68	0.5
Height (cm)	159.57	8.69	160.87	7.55	0.26
Weight (kg)	64.31	11.62	64.47	14.5	0.17
BMI (kg/m^2^)	25.26	4.14	25.97	5.12	0.28
Body fat (%)	34.6	8.29	34.04	8.53	0.39

Independent *t* tests were used for statistical analyses.

**Table 2:** Descriptive statistics between two groups.

	Total	BTE	IMT	
	*n* (%)	*n* (%)	*n* (%)	*p* Value
Participant	60 (100%)	30 (50%)	30 (50%)	
Sex ^b^				0.38
Male	15 (25%)	8 (26.7%)	7 (23.3%)	
Female	45 (75%)	22 (73.3%)	23 (76.7%)	
Age ^a^				0.5
40–49 years	13 (21.6%)	7 (23.3%)	6 (20%)	
50–59 years	25 (41.7%)	10 (33.3%)	15 (50%)	
60–65 years	22 (36.7%)	13 (43.3%)	9 (30%)	
Asthma severity ^a^				0.5
Moderate	56 (93.3%)	28 (93.3%)	28 (93.3%)	
Severe	4 (6.7%)	2 (6.7%)	2 (6.7%)	

^a^: Independent *t* test (*p* < 0.05); ^b^: Pearson chi-square test (*p* < 0.05).

**Table 3:** Comparison of spirometry, clinical symptoms, and physical activity between breathing exercises (BTE) and inspiratory muscle training (IMT) group.

Outcome	Groups	Time Period					*p* Values		
		Pre	95% CI	Post	95% CI	Δ	“Group” × “Time”	“Time”	“Group”
Pulmonary functions									
FVC (% predicted)	BTE	87.05 ± 15.3	81.82–92.28	85.62 ± 14.75	80.78–90.46	−1.43 ± 11.56 ^†^	0.045 *	0.312	0.147
	IMT	79.38 ± 13.26	74.15–84.62	83.63 ± 11.54	78.79–88.47	4.25 ± 9.82 ^†^			
FEV1 (% predicted)	BTE	82.77 ± 17.19	76.73–88.81	82.37 ± 16.56	77–87.74	−0.4 ± 6.34	0.263	0.498	0.505
	IMT	79.12 ± 15.84	73.08–85.17	80.73 ± 12.55	75.36–86.1	1.61 ± 7.36			
Respiratory muscle strength									
PImax (% predicted)	BTE	82.45 ± 32	71.69–93.22	99.38 ± 35.03	88.25–110.5	16.92 ± 12.94 ^†^	0.00 *	0.00 *	0.527
	IMT	71.19 ± 26.66	60.42–81.95	101.03 ± 25.04	88.9–112.16	29.84 ± 13.16 ^†^			
PEmax (cmH_2_O)	BTE	53.87 ± 20.72	45.51–62.23	70.67 ± 25.5	61.39–79.94	16.8 ± 15.52	0.9	0.00 *	0.918
	IMT	54.7 ± 24.83	46.34–63.06	71.07 ± 25.26	61.79–80.34	16.37 ± 13.67			
Asthma control									
ACT	BTE	21.43 ± 2.06	20.5–22.36	22.06 ± 1.86	21.34–22.79	0.63 ± 1.49	0.437	0.001 *	0.927
	IMT	21.3 ± 2.95	20.37–22.23	22.3 ± 2.1	21.58–23.03	1 ± 2.03			
ACQ	BTE	1.15 ± 0.44	0.98–1.33	1.02 ± 0.43	0.87–1.17	−0.13 ± 1.37	0.466	0.00 *	0.883
	IMT	1.2 ± 0.52	1.03–1.38	1 ± 0.41	0.85–1.16	−0.2 ± 0.31			
Functional capacity									
6MWT									
Distance (meters)	BTE	520.17 ± 74.8	493.35–546.98	545. ± 71.44	520.94–569.26	24.93 ± 42.03	0.237	0.00 *	0.741
	IMT	508.67 ± 71.92	481.85–535.48	545.06 ± 60.3	520.9–569.22	36.4 ± 31.45			
Heart rate (beats/min)	BTE	108.23 ± 13.8	103.02–113.45	109.7 ± 16.14	104.61–114.79	1.47 ± 16.39	0.318	0.076	0.524
	IMT	104.36 ± 14.72	99.15–109.58	109.53 ± 11.29	104.44–114.62	5.17 ± 11.66			
Blood oxygen saturation (%)	BTE	94.86 ± 1.83	94.18–95.55	94.16 ± 1.93	93.48–94.85	−0.7 ± 1.86	1	0.003 *	0.440
	IMT	94.53 ± 1.90	93.85–95.22	93.83 ± 1.82	93.15–94.52	−0.7 ± 1.6			
Physical activity									
3-D PAL (Kcal/kg/day)									
Retired	BTE	38.15 ± 5.67	35.33–40.98	39.51 ± 5.06	36.8–42.24	1.36 ± 2.29	0.587	0.001 *	0.765
	IMT	37.36 ± 3.95	34.43–40.31	39.15 ± 4.37	36.32–41.99	1.33 ± 2.53			
Employed	BTE	44.01 ± 4.57	40.76–47.28	45.34 ± 8.07	41.98–48.71	1.79 ± 1.5	0.462	0.001 *	0.467
	IMT	45.3 ± 8.06	42.14–48.48	47.33 ± 8.51	44.06–50.6	2.02 ± 2.65			

Data are expressed as mean ± standard deviation. * *p* < 0.05, significant differences. ^†^ *p* < 0.05, significant differences between intervention groups. 95% CI: 95% confidence interval; Δ: pre-post change value; Pre: pretest; Post: posttest; 6MWT: 6-min walk test; FVC: forced vital capacity; FEV1: forced expiratory volume in one second; PImax: maximal inspiratory pressure; PEmax: maximal expiratory pressure; ACT: asthma control test; ACQ: Asthma Control Questionnaire; 3-D PAL: three-day physical activity log.

## Data Availability

Data sharing is not applicable to this article.
